# The role of brain creatine in behavioral health conditions

**DOI:** 10.3389/fpsyt.2025.1667639

**Published:** 2025-11-07

**Authors:** Ellie S. Han, James R. Yancey, Deborah A. Yurgelun-Todd, Douglas G. Kondo, Danielle J. Boxer, Perry F. Renshaw

**Affiliations:** 1 Diagnostic Neuroimaging, Huntsman Mental Health Institute, Salt Lake City, UT, United States; 2 Department of Psychiatry, University of Utah School of Medicine, Salt Lake City, UT, United States; 3 Department of Psychiatry, Rocky Mountain Mental Illness Research, Education and Clinical Center (MIRECC), Salt Lake City Veterans Affairs (VA) Medical Center, Salt Lake City, UT, United States

**Keywords:** creatine, major depressive disorder, substance use disorder, posttraumatic stress disorder, anxiety disorder

## Abstract

Creatine, as a naturally occurring organic compound, has gained attention for its potential role in psychiatric health. The creatine kinase-phosphocreatine energy buffer system plays a crucial role in maintaining energy supply in the brain. Brain bioenergetic deficits, particularly those related to mitochondrial dysfunction, plays a critical role in the pathophysiology of psychiatric illnesses. A growing body of literature has focused on the potential therapeutic role of creatine supplementation in psychiatric illnesses. This review summarizes findings from preclinical, epidemiological, clinical and neuroimaging studies to examine creatine’s role as both a biomarker and therapeutic agent in psychiatric disorders, including Major Depressive Disorder, Anxiety Disorders, Posttraumatic Stress Disorder, and Substance Use Disorder.

## 1 Introduction

Over one billion people worldwide are affected by mental and addictive disorders (1). These conditions place a heavy burden on individuals as the leading causes of disability and premature death (2). In addition, psychiatric disorders have the strongest effect on suicide rates throughout the life course, with disorders including depression and substance use disorders increasing the risk of completed suicide by more than three times (3). Economically, disability and premature mortality due to mental disorders are associated with a global loss exceeding $4.7 trillion USD (2). Therefore, it is imperative that mental health be prioritized as a global health issue, with increased attention to effective and accessible treatment. Moreover, advancing the understanding of the underlying biological mechanisms of psychiatric disorders is crucial improving treatment.

Traditionally, behavioral health disorders were primarily understood through the monoamine theory, which stated that deficiencies in neurotransmitters such as serotonin and dopamine were the root cause of these disorders (4). However, there is now a growing focus on mitochondrial dysfunction as a contributing factor to psychiatric illnesses (5). The brain, with its high energy demand and large numbers of mitochondria, is particularly vulnerable to disruptions in mitochondrial ATP production (6). Previous studies have established that brain bioenergetic deficits – particularly those related to mitochondrial dysfunction – play a critical role in the pathophysiology of psychiatric illnesses. Mitochondria are implicated in several proposed mechanisms underlying psychiatric illnesses such as inflammation, oxidative stress, ferroptosis, etc., – all deeply interconnected and central to the development of psychiatric illnesses (7). In summary, mitochondrial dysfunction is a transdiagnostic pathophysiology across psychiatric illnesses such as Major Depressive Disorder (MDD), Anxiety Disorders, Posttraumatic Stress Disorder (PTSD), or Substance Use Disorder (SUD) (8–11).

Creatine (Cr) is a nitrogenous organic compound produced endogenously mostly in the kidneys and liver from arginine and glycine (12, 13). Human beings synthesize about 50% of necessary creatine and supplement the remaining 50% through diet (13). Approximately 95% of creatine is stored in skeletal muscles, while the remaining 5% is found in bone tissue and the brain (14). Although research on creatine supplementation has primarily focused on muscle function, creatine also plays a crucial role in maintaining adenosine triphosphate (ATP) supply in the brain, especially during times of high demand (10) such as hypoxia (15), sleep deprivation (16, 17), mental fatigue (18) or possibly in psychiatric illnesses that involve brain bioenergetic deficits (8). Additionally, creatine supports neurons that require high amounts of ATP for various cellular processes, including learning, memory, energy homeostasis and mitochondrial function (19).

In the brain, creatine plays an essential role in the storage and transmission of energy, as part of the creatine kinase-phosphocreatine (PCr) system (20). The enzyme creatine kinase catalyzes the reversible exchange of a phosphate group between Cr and ATP that manages the energy homeostasis in the body (14).


$$Cr+ATP↔PCr+ADP+\;H^{+}$$

The creatine-kinase-PCr system serves as a buffer for ATP homeostasis when rates of synthesis are greater than the rates of consumption and stores energy as PCr. When more energy is needed, PCr is efficiently transported from sites of ATP synthesis to sites of ATP break down and expenditure (21). More specifically, ATP is synthesized in the mitochondria via oxidative phosphorylation. Within the mitochondrial intermembrane space, mitochondrial creatine kinase facilitates the transfer of a phosphate group from ATP to creatine, producing phosphocreatine. The resulting PCr then diffuses into the cytosol, where it functions as a mobile, high-energy phosphate reservoir (22). This system is highly efficient: ATP can be synthesized from PCr 12 times faster than oxidative phosphorylation and more than 70 times faster than *de novo* pathways (23). Therefore, the creatine-kinase-PCr system plays a vital role in ensuring that neurons have sufficient energetic reserves to meet the demands of healthy brain function.

For more than a decade, creatine has been one of the most popular dietary supplements worldwide for its efficacy in enhancing exercise performance and improving symptoms of neuromuscular and cardiometabolic diseases. Although research on creatine has traditionally investigated its role in skeletal and muscle energetics, a growing body of evidence now supports its mechanistic potential to exert therapeutic and neuroprotective effects in brain health (24). Building on findings that phosphocreatine serves as a critical energy reservoir in the brain, and recognizing that many psychiatric disorders are characterized by impaired brain energy metabolism, researchers have begun to examine creatine as a potential adjunctive treatment in psychiatric illnesses. For example, animal models have established creatine’s anti-depressant-like effects, especially in female rats and in combination with antidepressants (25–27). Furthermore, preclinical models have reported reduced brain creatine levels with methamphetamine (MA) administration and exposure to stress (28–31). Given its critical role in cellular energy metabolism, creatine is intimately involved in mitochondrial function and has become of increasing interest in psychiatric research. These findings provide rationale for further investigating creatine supplementation as a potential strategy to enhance psychiatric health in human beings.

Oral creatine supplementation has been shown to increase creatine levels in the human brain and increase the PCr/ATP ratio, with regions of initially decreased PCr levels showing most increase in PCr (32) when measured by proton or phosphorous magnetic resonance spectroscopy (1H or 31P-MRS) (10, 14). MRS is a neuroimaging technique that allows investigators to measure brain chemistry and *in vivo* metabolism non-invasively, using the magnetic spin property of odd-mass numbered atoms such as 1H or 31P (33). This allows investigators to observe biochemical processes in the body, including the brain. Lyoo and colleagues conducted a placebo controlled MRS study in which healthy subjects who took creatine monohydrate at 0.3 g/kg/day for seven days and 0.03 g/kg/day the following week, exhibited significantly increased brain creatine levels compared to the placebo group (Cr/NAA d=1.67, Cr/Cho d=0.93). Additionally, participants in the creatine group showed increased product PCr (d=0.51) and decreased substrate beta-NTP (d=-0.62). Study investigators concluded that these changes were indicative of changes in brain energy metabolism after oral creatine supplementation (34).

A growing body of literature has examined the potential therapeutic role of creatine supplementation in psychiatric disorders. Allen (2012) provided a comprehensive overview of the neurobiology underlying the phosphocreatine energy system and its relevance to psychological stress, schizophrenia, and mood and anxiety disorders (35). Kious et al. (2019) concentrated specifically on creatine supplementation for the treatment of depression (36). More recently, Forbes et al. (2022) investigated the effects of creatine, and explored guanidinoacetic acid supplementation as an alternative to or adjunctive with creatine supplementation, exploring implications for neurological and mental health conditions (14). These reviews represent a subset of the ongoing research in this area. Given the expanding interest in this field, the present review aims to evaluate the role of creatine supplementation in neuropsychiatric conditions such as MDD, Anxiety Disorders, PTSD, and SUD with a particular focus on its potential as an adjunctive treatment in behavioral mental health care.

## 2 Methodology

For the current review, key terms like “creatine AND depression,” “creatine AND substance use disorder,” and “creatine AND PTSD” were used to search relevant literature on PubMed and Cochrane Library databases. Although most studies cited in this review were published within 2009-2025, due to the limited number of literatures on creatine supplementation in psychiatric illnesses, some studies dating further back were included. Therefore, this review puts greater emphasis on studies published during the last two decades, given that the field has been building on this line of research overtime. The above search yielded 2708 records: 2506 articles from PubMed and 202 articles from Cochrane Library. All the records were title/abstract screened. Only those articles directly related to creatine supplementation in depression, substance use disorders, and PTSD were included in the current review. In contrast, records that discussed creatine in context of physical health conditions, those not available in the English language, and whose full text was unavailable were excluded. The findings of the review were described narratively.

### 2.1 Depression and anxiety + creatine

Preclinical models have provided initial support for the antidepressant potential of creatine. For example, Allen et al. assessed effects of combining creatine supplementation with low-dose fluoxetine treatment for four weeks on depression-like behaviors using the forced swim test in male and female rats. Female rats fed a 4% creatine diet exhibited antidepressant-like behaviors with or without fluoxetine, while male rats did not show such effects. When fluoxetine was administered, female rats receiving creatine supplementation displayed enhanced antidepressant-like responses compared to those treated with fluoxetine alone. Estrous cycle data suggested that ovarian hormones in female rats may mediate these antidepressant effects in females (35).

Epidemiological studies further support a link between creatine metabolism and depressive symptoms. An analysis of 22,692 participants from the National Health and Nutrition Examination Survey (NHANES) found an inverse association between dietary creatine intake and depression, particularly among women (d=-0.25), individuals aged 20-39 (d=-0.34), and those not taking antidepressant or anxiolytic medications (d=-0.27) (37). Additionally, data from the China Health and Retirement Longitudinal Study found that compared to participants with high creatinine levels (the breakdown product of creatine phosphate), those with moderate and low levels of serum creatine had higher risk of depression (middle level: OR = 1.41, 95% CI = 1.26-1.57; low level: OR = 1.67, 95% CI = 1.49-1.88) (38).

Neuroimaging studies using MRS have corroborated these findings. Kato et al. (1992) reported significantly reduced PCr levels in individuals with severe depressive symptoms compared to those with milder symptoms (d=-1.05). Furthermore, beta-NTP, a 31P MRS proxy measure of energy homeostasis, was significantly lower in female subjects, highlighting possible sex differences in bioenergetic dysfunction in MDD (39). These biological differences align with broader epidemiological patterns showing that women are diagnosed with MDD at higher rates and at earlier ages than men, with differing symptom profiles across sexes (40, 41).

Anxiety disorders and MDD co-occur in high prevalence and have negative psychosocial and medical impacts (42). Therefore, it is important to note that anxiety disorders demonstrate similar patterns of altered brain bioenergetics. For instance, Yue et al. reported that individuals with anxiety disorders had reduced creatine concentrations in the left dorsolateral prefrontal cortex (d=0.82). Creatine concentrations negatively correlated with scores on the Liebowitz Social Anxiety Scale, indicating that lower creatine levels were associated with more severe social anxiety symptoms such as avoidance (r=-0.589) and fear (r=-0.553) (43). Similarly, individuals with General Anxiety Disorder (GAD) without a history of early trauma exhibited lower levels of total creatine (creatine + phosphocreatine) compared to healthy controls (left d=1.17, right d=1.44). GAD patients with a history of early trauma, however did not show significant differences compared to healthy controls (44). Collectively, these findings suggest that bioenergetic dysregulation, particularly in the brain creatine levels, may serve as a biomarker of psychiatric illness.

Importantly, baseline brain creatine levels may also predict treatment response. Individuals with higher baseline PCr levels tend to respond more favorably to selective serotonin reuptake inhibitors (SSRIs), including escitalopram and fluoxetine (26, 45). Therefore, creatine augmentation with standard antidepressants may lead to an earlier and greater response to standard antidepressant treatment (45). Roitman et al. observed that in eight patients with unipolar treatment-resistant depression (TRD), a four-week course of 3–5 g/day of adjunctive creatine supplementation led to significant improvements in clinically relevant depression and anxiety measures. For example, the Hamilton Depression Rating Scale (HAM-D) scores decreased from 23.14 at baseline to 12.57 at week 4 (d=1.67), indicating a significant decrease from the clinically moderate depression category on the scale to the mild depression category. Additionally, the Hamilton Anxiety Scale (HAS) scores decreased from 18.71 at baseline to 12.00 at week 4 (d=1.37), also indicating a significant improvement from the mild to moderate anxiety category to the mild anxiety category. Clinical Global Impression scores also decreased from 4.43 at baseline to 3.00 at week 4 (d=2.86) (46). Similarly, an eight-week double-blind randomized clinical trial conducted by Lyoo et al. demonstrated that creatine augmentation accelerated improvements in HAM-D scores in escitalopram-treated females with depression. Those receiving creatine exhibited significant improvement compared to the placebo group, as early as two weeks after treatment initiation (odds ratio=11.68), and these gains were maintained through week eight (odds ratio=6.92) (45). A complimentary study by Kious et al. investigated the effects of augmenting conventional antidepressants with creatine and 5-hydroxytryptophan (5-HTP) in women with SSRI or Serotonin-Norepinephrine Reuptake Inhibitor (SNRI)-resistance. The study reported a significant reduction in depressive symptoms, with a mean 60% decrease in HAM-D scores (d=3.19) (47). However, it is also important to note the effects of the dosage and duration of creatine administration. For instance, Nemets and Levine did not see any significant benefits from creatine augmentation at low doses (5-10g) for four weeks (48), underscoring the need for either higher dosages (20 grams or higher) over shorter durations or sustained low-dose regimens lasting at least eight weeks to alleviate symptoms of MDD (43).

New research on creatine has also shown potential as an adjunct to psychological therapies. Sherpa et al. augmented Cognitive Behavioral Therapy (CBT) with creatine in patients with MDD (49) and found that the combined intervention group showed lower scores on the Patient Health Questionnaire-9 (PHQ-9) (d=-2.11), a depression symptom measure (50), compared to those receiving CBT with placebo (51). These results raise compelling questions about how improving brain bioenergetics can enhance cognitive mechanisms engaged in psychotherapy. Together, the data suggest that creatine may improve outcomes even in the absence of pharmacotherapy and could be integrated across treatment modalities for TRD (52).

The need for novel treatment options is especially urgent in adolescents with MDD. Adolescents experience more recurrent episodes, higher suicidality, and increased hospitalization compared to adults with MDD (53). Importantly, at least 40% of adolescents fail to respond to first-step interventions (54, 55), and while emerging interventions such as ketamine and electroconvulsive therapy show promise, their use in adolescent populations remains limited (56). Thus, there is a critical need for novel, safe and effective antidepressant agents in adolescents.

To begin addressing this need, Kondo et al. treated five female adolescents with SSRI-resistant MDD with a combination of fluoxetine and 4 g/daily of creatine over 8 weeks. Following creatine augmentation, subjects’ Children’s Depression Rating Scale-Revised (CDRS-R) declined 56% (d=4.21), and compared to the healthy controls, those who received creatine showed a significant increase in brain PCr concentration after eight weeks of daily creatine augmentation (d=0.33) (57). The CDRS-R serves as a reliable and valid metric for adolescents with depression (58). In a follow-up dose-ranging trial, adolescent females receiving 10 g/day of creatine showed a 9.1% increase in frontal lobe PCr levels, whereas the placebo group experienced a slight decline of 0.7% (d=0.8) (52). These results mirror adult findings and reinforce the hypothesis that creatine improves brain bioenergetics through the elevation of high-energy phosphate stores.

In summary, the evidence suggests that creatine supplementation may offer a safe and biologically plausible adjunct to traditional treatments for MDD, with particular relevance for women. Given the consistent findings across animal models, epidemiological data, MRS studies, and clinical trials, future research should continue to explore sex-specific mechanisms and optimize dosing protocols to harness the full therapeutic potential of creatine in psychiatric care.

### 2.2 PTSD + creatine

Approximately 70% of the adult population worldwide has experienced at least one traumatic life event (59), and lifetime prevalence of Posttraumatic Stress Disorder (PTSD) is 6.1% (60), indicating individual differences in stress recovery from traumatic life events. As discussed above PTSD is highly comorbid with depression and both disorders may share underlying neurobiological mechanisms including reactivity of the HPA axis (61) and brain mitochondrial dysfunction (9). Emerging research has focused on how brain creatine may be implicated in the brain’s response to stressful events. Preclinical models suggest that brain creatine levels, particularly in the frontal brain regions, is reduced when exposed to stress (31). For example, subordinate animals exposed to psychosocial defeat by dominant animals exhibit significantly less total creatine (creatine + phosphocreatine), along with reduced hippocampal volume and impaired neurogenesis (35).

In patients with PTSD, trends toward reduced creatine levels have been observed throughout the dorsal anterior cingulate cortex (ACC) (62, 63), hippocampus and occipital white matter (64, 65). In addition, in the ACC, lower creatine concentrations correlated with higher arousal scores, suggesting that prefrontal deficits in brain bioenergetics of the prefrontal tissue is associated with hyperarousal observed in PTSD (63, 66). This information points to the need to further explore creatine levels in trauma response and stress recovery. Another study by Yancey et al. used 1H-MRS to measure creatine levels in US Veterans who experienced at least one traumatic life event, they found that higher total creatine (creatine + phosphocreatine) in the anterior cingulate was correlated with better traumatic recovery as assessed via retrospective self-report (r(25)=0.43) (31). This finding suggests that creatine may be associated with greater stress recovery. The similarity between findings from the preclinical animal models that suggest that creatine concentrations are reduced following exposure to high-stress in the laboratory conditions (67), together with the findings from clinical studies that higher levels of total creatine were associated with greater self-reported stress recovery, suggests that creatine may be an important factor related to capacity for responding to and recovering from stressful environmental conditions (31). Given creatine’s association with MDD and PTSD symptoms, more research is necessary to investigate the underlying mechanism of creatine and its interaction with various psychiatric illnesses.

Regarding creatine as a possible therapeutic agent, there are successful cases of creatine augmentation in PTSD patients such as this case of a 52-year-old woman who suffered from PTSD and comorbid depression and fibromyalgia, after losing her left eye in a terror bombing scene. This patient showed resistance to standard psychotherapy. When the patient was treated with creatine for 4 weeks (3 g daily in the first week, then 5 g daily) with continued ongoing psychotropic treatment, the patient showed improvements in depression and fibromyalgia symptoms, with reported improved sleep patterns and somatic symptoms, leading to a 30% increase in her quality of life (68). This patient was part of an open-label clinical study conducted by Levine et al. that carried out creatine augmentation to ongoing psychotropic treatment in treatment-resistant PTSD patients. In the creatine group, Clinical Global Impressions scores, used to measure general psychiatric health (69), improved significantly (d=0.47). In addition, in the creatine group, all clinician-administered PTSD scales parameters mildly improved during the study (d=0.57), with intrusiveness scores improving the most, and HAM-D scores also improving significantly (d=0.67). In particular, treatment-resistant PTSD patients with comorbid depression showed greatest improvements from creatine augmentation (70). The positive and hopeful results of this study provide further evidence for creatine augmentation in PTSD treatment.

### 2.3 Substance use dual-diagnosis + creatine

Substance use disorders (SUD) are frequently comorbid with MDD and PTSD. 11-41% of individuals seeking treatment for SUD meet diagnostic criteria for PTSD, a comorbidity associated with more severe cravings and higher relapse rates compared to SUD alone (71). Furthermore, both PTSD and SUD independently increase the risk for developing MDD (71). When MDD co-occurs with either PTSD or SUD, individuals experience more severe psychosocial impairments than with either condition alone (71). Adolescents with SUD are more likely to have MDD than those without (72) and patients diagnosed as having both depression and SUD tend to have more severe clinical courses and worse outcomes than those who only have either or (73). A meta-analysis by Stokes et al. has also raised concerns that SSRI treatments, either alone or in combination with relapse prevention medications in SUD, such as naltrexone, had no significant effect on depressive symptoms in people with MDD and comorbid addictions (74).

Methamphetamine Use Disorder (MUD), in particular, presents distinct clinical challenges due to its profound neurotoxic, psychiatric, and medical consequences. MUD is one of the most addictive and treatment-resistant forms of SUDs, which results in serious impairments in social and occupational functioning (75). MUD can develop rapidly, and is characterized by a cyclical pattern of intense use followed by intermittent abstinence. Medically, it is associated with severe cardiovascular and cerebrovascular complications, which constitute the leading causes of mortality in MA users (76). Neurotoxic effects in MUD are also prominent: in a study involving 100 adults with MUD and no medical comorbidities, 36% exhibited psychiatric comorbidities such as mood disorders and anxiety disorders, and 25% of those were substance induced (77). In addition, MUD is difficult to treat – those who do not engage in treatment show a 5-year remission rate of 30%, and even among those who do receive treatment, 61% relapse within the first year (76). Despite the urgent need for effective treatments, pharmacological options for MUD remain limited and underdeveloped (78).

Numerous clinical trials investigating medications for MUD have yielded mixed or inconclusive results. For example, bupropion was found in some studies to reduce cravings but not in others (79, 80). Similarly, naltrexone has been shown to modify MA cravings, possibly via endogenous opioid pathways (81); however, evidence does not currently support its efficacy in promoting abstinence or reducing relapse rates (82). Other medications such as vigabatrin, ondansetron, topiramate, and gabapentin are under investigation, but their efficacy for MUD has not yet been established (83). As of now, no FDA approved pharmacological treatment is available for MA dependent individuals seeking treatment in the United States (83, 84). Therefore, further research into medications for MUD treatment is necessary.

As with depression, anxiety, and PTSD, there is growing evidence that mitochondrial dysfunction and brain energy metabolism play key role in the pathophysiology of MUD (35). In preclinical models, MA use has been associated with reduced activity in the electron transport chain (ETC), particularly in striatal regions, leading to decreased ATP production (28, 29). Preclinical studies in rats have demonstrated MA-induced reductions in ETC complexes I, II, and III, which contribute to impaired mitochondrial function and decreased energy production (30).

Consistent with these preclinical findings, human studies have reported altered brain energy metabolism in MA users. Relative to healthy controls, MA users exhibited significantly reduced levels of total creatine (PCr + creatine) in the frontal lobe as measured by 1H-MRS (d=-0.71) (85). Additionally, 31P-MRS data revealed sex differences, with significantly lower phosphocreatine-to-total phosphorous (PCr/TPP) ratios in females MA users than male users, despite lower daily amounts of MA (d=-1.0) (11, 85). This finding points to the possibility that along with mitochondrial dysfunction observed in MA dependence (28–30), decreased brain PCr high energy phosphate reserve in MA users may also contribute to the reduced brain energy metabolism (86), especially in female MA users.

Following this line of investigation, Hellem et al. conducted a pilot study administering 5g of creatine monohydrate daily to 14 female participants with comorbid depression and MA dependence over an eight-week period (11). Post-treatment assessments showed significant increases in brain PCr concentrations as measured by 31P-MRS (d=0.92). Concurrently, participants exhibited significant reductions in depressive symptoms (HAM-D) (d=1.99) and anxiety (Beck Anxiety Inventory) (d=1.71), along with a 50% decrease in MA positive urine drug screens by week six (11). These preliminary findings suggest that creatine may be a promising adjunctive treatment for co-occurring depression and MA dependence. However, despite positive results, there is a lack of literature focusing on creatine’s potential as an adjunctive treatment for MUD, and furthermore, SUD. Therefore, further research is necessary to understand creatine’s treatment efficacy in SUD, especially in women, and to elucidate the mechanisms underlying interaction behind creatine in SUD and co-occurring mood disorders.

## 3 Conclusion

Creatine is an organic acid endogenous to mammals that plays a key role in supporting brain bioenergetics. It can also be readily assessed *in vivo* using MRS. As discussed in this review, variations in brain creatine have been identified across depression and PTSD – two psychiatric disorders with substantial public health implications. Variations in creatine have also been observed among patients with methamphetamine addiction. The fact that creatine levels are related to various mental health conditions suggest that it could be a potential role as a transdiagnostic marker of impaired brain bioenergetic capacity and a proxy marker of mitochondrial (dys)function. This idea would be consistent with increasing evidence across scientific modalities that mitochondrial function plays an important role in mental health.

Moreover, as discussed above, brain creatine can be inexpensively supplemented with creatine monohydrate supplementation, and it has an excellent safety profile. Thus, creatine holds promise not only as putative biomarker, but also treatment target across neuropsychiatric conditions. Indeed, the review of this literature suggests that creatine has shown efficacy as an adjunctive to SSRI treatment for TRD MDD, particularly in female patients. Importantly, clinical trials utilizing MRS have shown that changes in brain phosphocreatine corresponded with treatment responses, providing strong mechanistic evidence that creatine supplementation addresses symptoms by altering brain bioenergetic resources. Though more limited, there is also evidence that creatine supplementation may also be effective as an adjunctive to pharmacotherapy for PTSD, and that it may improve symptoms of methamphetamine use disorder. However, more clinical trials, particularly those with MRS are needed to further validate these encouraging initial findings.

Recently, exciting work has shown that it can be combined with talk therapy to improve depressive symptoms. This finding may be especially relevant for PTSD as evidence-based psychotherapies (e.g. Prolonged Exposure and Cognitive Processing Therapy) remain the gold-standard treatments. Future research is needed to explore the mechanisms by which creatine may affect cognitive and affective processes that are engaged in psychotherapy. Overall, brain bioenergetics and underlying mitochondrial function is becoming increasingly recognized as an important biological contributor to psychiatric illness. Our review provides an overview of creatine both as an *in vivo* marker of brain bioenergetic health and as potential therapeutic target across a range of conditions. Future research is necessary to better understand the promise of creatine supplementation as both an adjunctive and monotherapy.

Finally, the relevance of creatine to the development of novel behavioral health interventions is underscored by the current enthusiasm in the field surrounding ketamine and the serotonergic psychedelics. These repurposed drugs are thought to have “transformative” potential in the treatment of neuropsychiatric disorders (87), and are described as exemplars of the “disruptive psychopharmacology” that is destined to inform the next generation of psychotropic medications (88). It is therefore notable, that a series of preclinical experiments has demonstrated that creatine and ketamine produce their antidepressant-like effects in animal models through the same mechanism (89, 90). This mechanistic pathway, known as the mammalian target of rapamycin (mTOR), is the downstream mechanism-of-action of both ketamine and the serotonergic psychedelics, according to recent work from the U.S. National Institute of Mental Health (91). Moreover, creatine supplementation upregulates mTOR signaling (92), and ketamine administration increases brain creatine kinase system activity (93). However, further research to elucidate the direct causal mechanism by which mitochondrial ATP and mTOR signaling contribute to psychiatric illnesses. In particular, we propose that monitoring these processes through MRS, especially 31P-MRS, will provide valuable insights. Further work is needed to elucidate the interactions and overlap between creatine and the dissociative antidepressants, which ensure creatine’s place as a mechanistic biomarker and potential treatment intervention in behavioral health conditions.

The limitations to the extant literature at the intersection of creatine and behavior disorders must be acknowledged. These include the heterogeneity in study design and statistical analyses, the limited sample sizes, the representativeness of the study populations recruited in creatine clinical trials, the observational nature of data reported in many publications, and the recall bias and interindividual variability that are unavoidable features of the self-report measures that have traditionally served as primary outcome variables in mental health research. The latter has motivated the National Institute of Mental Health to transition toward “mechanistic” clinical trials, in its investigator-initiated funding announcements. This move is designed to facilitate the identification of the mechanism(s) by which pharmacological, psychosocial, and neuromodulatory interventions produce objectively measurable change(s) in research participants. Like many investigational treatments, the mechanism of creatine has yet to be elucidated. Converging lines of evidence suggest that the mechanism of mental disorders may be related to impaired energy metabolism in brain, and offer directions for future creatine work. These include analysis of mitochondria-related genes (94), mitochondrial dynamics (95), and. While converging lines of evidence implicate mitochondria in psychiatric disorders (96), the process of defining the precise mechanism of mental health treatments, including creatine, is complicated by the fact that the neurobiology of psychiatric illness itself remains unknown (97) – leaving mitochondrial dysfunction to be described as “the missing link” (95). One newly-developed tool is the MitoBrainMap v1.0 (98), which offers the possibility of understanding the connection between regional mitochondrial activity and neurocognitive function, thus opening novel possibilities for human medicine and research (98). If this and other new technologies can be integrated with existing neuroimaging methods (99), the health of brain mitochondria can be serially measured in response to a specific treatment. Thus, while the field has just entered the era of mechanistic clinical trials, the prospect of defining the neural substrates of mental illness, and identifying relevant treatment targets, appears to be at hand.

Overall, although existing small-scale, open-label, and pilot studies provide valuable preliminary guidance, there remains a crucial need for larger, well-designed clinical trials. Many of the current studies share limitations in design and sample size, which introduces variability across findings. There is especially variability by condition: large-scale double-blind clinical trials provide relatively strong support for depressive disorders, whereas in other psychiatric illnesses the evidence is more limited but still promising. Future research should expand upon these early findings by employing larger sample sizes and more rigorous methodologies to elucidate the mechanisms of creatine, and its efficacy in improving psychiatric illnesses.
